# Nano-risk Science: application of toxicogenomics in an adverse outcome pathway framework for risk assessment of multi-walled carbon nanotubes

**DOI:** 10.1186/s12989-016-0125-9

**Published:** 2016-03-15

**Authors:** Sarah Labib, Andrew Williams, Carole L. Yauk, Jake K. Nikota, Håkan Wallin, Ulla Vogel, Sabina Halappanavar

**Affiliations:** 1 Environmental Health Science and Research Bureau, Health Canada, Ottawa, ON K1A 0K9 Canada; 2 National Research Centre for the Working Environment, Lerso Parkallé 105, DK-2100 Copenhagen, Denmark; 3 Department of Public Health, University of Copenhagen, DK-1353 Copenhagen K, Denmark; 4 Department of Micro- and Nanotechnology, Technical University of Denmark, DK-2800 Kgs Lyngby, Denmark

**Keywords:** Nano, Risk assessment, Toxicogenomics, Adverse outcome pathways, Benchmark dose, Case study, Lung fibrosis

## Abstract

**Background:**

A diverse class of engineered nanomaterials (ENMs) exhibiting a wide array of physical-chemical properties that are associated with toxicological effects in experimental animals is in commercial use. However, an integrated framework for human health risk assessment (HHRA) of ENMs has yet to be established. Rodent 2-year cancer bioassays, clinical chemistry, and histopathological endpoints are still considered the ‘gold standard’ for detecting substance-induced toxicity in animal models. However, the use of data derived from alternative toxicological tools, such as genome-wide expression profiling and in vitro high-throughput assays, are gaining acceptance by the regulatory community for hazard identification and for understanding the underlying mode-of-action. Here, we conducted a case study to evaluate the application of global gene expression data in deriving pathway-based points of departure (PODs) for multi-walled carbon nanotube (MWCNT)-induced lung fibrosis, a non-cancer endpoint of regulatory importance.

**Methods:**

Gene expression profiles from the lungs of mice exposed to three individual MWCNTs with different physical-chemical properties were used within the framework of an adverse outcome pathway (AOP) for lung fibrosis to identify key biological events linking MWCNT exposure to lung fibrosis. Significantly perturbed pathways were categorized along the key events described in the AOP. Benchmark doses (BMDs) were calculated for each perturbed pathway and were used to derive transcriptional BMDs for each MWCNT.

**Results:**

Similar biological pathways were perturbed by the different MWCNT types across the doses and post-exposure time points studied. The pathway BMD values showed a time-dependent trend, with lower BMDs for pathways perturbed at the earlier post-exposure time points (24 h, 3d). The transcriptional BMDs were compared to the apical BMDs derived by the National Institute for Occupational Safety and Health (NIOSH) using alveolar septal thickness and fibrotic lesions endpoints. We found that regardless of the type of MWCNT, the BMD values for pathways associated with fibrosis were 14.0–30.4 μg/mouse, which are comparable to the BMDs derived by NIOSH for MWCNT-induced lung fibrotic lesions (21.0–27.1 μg/mouse).

**Conclusions:**

The results demonstrate that transcriptomic data can be used to as an effective mechanism-based method to derive acceptable levels of exposure to nanomaterials in product development when epidemiological data are unavailable.

**Electronic supplementary material:**

The online version of this article (doi:10.1186/s12989-016-0125-9) contains supplementary material, which is available to authorized users.

## Background

The increased production and use of engineered nanomaterials (ENMs) in industrial and consumer products has raised concerns about potential health and environmental risks posed by exposure to these materials. ENMs exhibit size-associated properties. While the chemical properties such as, solubility and chemical structure are traditionally known to influence the toxicity of bulk chemical substances, the toxicity of ENMs is influenced by their distinct physico-chemical properties, such as being insoluble (as opposed to soluble bulk chemicals), and having a small size, large surface area, and increased surface reactivity. Within the biological microenvironment, these unique properties allow ENMs to interact with surrounding biomolecules and structures in a complex and non-specific manner. As a result, ENMs are expected to be toxicologically distinct from soluble chemicals and more active than larger particles of similar chemical composition. Despite this knowledge, a validated framework for human health risk assessment (HHRA) of ENMs has yet to be established. Sustained development of nanotechnology-enabled products will be hampered by the lack of a pragmatic risk assessment paradigm that can effectively address the unique aspects of nanomaterial risk assessment in a timely manner.

Given the unique properties of ENMs, standardized toxicity testing methods developed originally for bulk chemicals were tested for their suitability for investigating ENM-induced effects [1–6]. Although, the Organization for Economic Co-operation and Development (OECD) has stated that in general the OECD guidelines developed for bulk chemicals may be applicable to ENMs, based on the types of endpoints studied, modifications and adjustments are needed in regards to the sample preparation and analysis. Consequently, significant efforts have been made to develop novel methods, or to modify existing in vivo and in vitro test methods, to assess ENM toxicity [7]. However, validation of these new methods has been challenging due to the lack of sufficient amount of reference materials and harmonized protocols. An additional challenge is the large number of ENMs requiring immediate human health hazard and risk assessment. Indeed, the generation of toxicological data for each ENM variant using conventional single end-point based methods is not practical. To circumvent this problem, a strategy has been proposed for ENMs that involves the use of high-content omics tools or in vitro high-throughput assays to examine the toxicity induced by well characterized ENMs in short-term toxicity models for screening and prioritization [8], a strategy that is in line with the United States National Research Council’s vision of a paradigm shift in toxicology [9].

Whole genome gene expression profiling allows for the simultaneous analysis of changes in the expression of all the genes in a tissue or cell type following substance exposure. Numerous studies have employed genomics methods, in combination with bioinformatics tools for ontological and pathway analyses, to investigate the underlying molecular mechanisms of action of toxicants [10–14]. These studies have demonstrated that global changes in gene expression following exposure to a substance are associated with the development of Labib toxicological phenotypes in a dose-dependent manner [15].

In our previous studies, we used whole genome gene expression profiling and bioinformatics analyses of mouse models to study the specific particle properties that influence toxicity and to elucidate the mechanisms of pulmonary response induced by various types of ENMs including: nano-titanium dioxide particles (nanoTiO2) [16–18], multiwalled carbon nanotubes (MWCNTs) [19, 20], and carbon black (CB) [21–23]. These studies demonstrated that the molecular perturbations at the gene expression and pathway level leading to lung inflammation following exposure to ENMs are the same regardless of the class of ENM studied; however, the magnitude (number of genes and the fold-change response) of the perturbation varies. Subsequently, we conducted a meta-analysis of gene expression data catalogued from 15 individual genomics datasets describing lung responses to disease-inducing chemical substances, pathogens, or ENMs in mouse models, and identified gene expression signatures predictive of disease-causing ENMs [24]. In another study, we used a combination of bi-clustering of gene expression data and gene set enrichment analyses methods to identify gene sets in ENM-induced gene expression changes that are similar to disease conditions [25]. The latter studies demonstrated that gene expression data can be used to predicting disease potential of ENMs before the phenotype is established, and therefore can be used to prioritize ENMs for further toxicological investigation. Thus, the of genomics tools to characterizing ENM-induced toxic responses is well established.

The use of genomics approaches to provide information on a chemical’s hazard and mode-of-action (MOA) is gaining acceptance in the regulatory community; however its utility in a regulatory context and a specific paradigm for genomics-driven quantitative HHRA has yet to be established. Approaches for quantitative dose–response analysis of genomics endpoints have been developed and methods to derive transcriptional benchmark dose (BMD) values that could be applied to quantitative risk assessment have been explored [26–29]. Although several case studies [26, 28, 30] have been conducted to demonstrate the potential use of gene expression data in deriving points of departure (PODs) for risk assessment, and guidance documents to describe the strategies and methods involved have been developed, much more work is needed before full integration of genomics in routine quantitative HHRA of toxic substances can be realized. In the interim, the value of genomics data as supplementary information contributing to the weight of evidence is recognized [31].

**Fig. 1:**
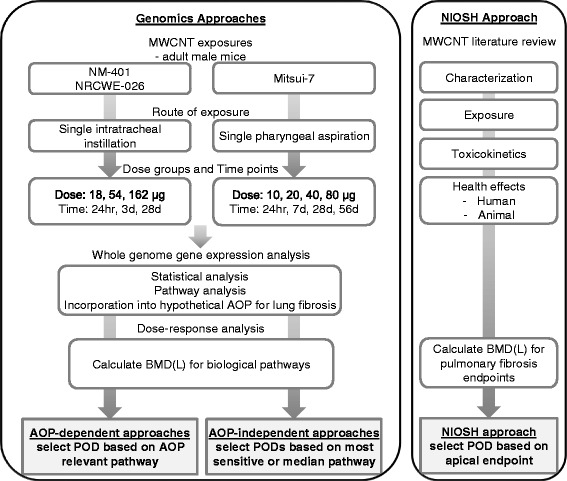
Comparison of traditional and genomics approaches for determining points of departure for exposure to MWCNT

In the present case study, we explored the use of toxicogenomics to establish PODs for MWCNT-induced lung fibrosis, and assessed its relevance for HHRA of MWCNTs. MWCNTs are widely used ENMs in industrial and biomedical sectors. Short-term and sub-chronic studies in rodents have shown that MWCNTs are bio-persistent and induce various effects in lungs, including persistent pulmonary inflammation, granulomas, and lung fibrosis, following exposure via inhalation, instillation, or pharyngeal aspiration [32–35]. Occupational exposure studies have estimated human CNT exposure levels of 10 – 300 μg/m^3^ in facilities such as research laboratories and production plants where CNTs are produced, processed, used, disposed of, or recycled [36–42] (reviewed in [43]). The United States National Institute for Occupational Safety and Health (NIOSH) recently completed a HHRA for CNT-induced lung fibrosis that takes into consideration existing toxicological data from animal models [43]. We used this published report to derive POD values for apical endpoints. For the derivation of genomics driven POD values, first, we used the framework of adverse outcome pathway (AOP) to logically and systematically organise the mechanistic information available in the literature concerning CNT-induced lung fibrosis and the results of global transcriptomic analyses conducted on mouse lungs exposed to three individual types of MWCNTs [19, 34, 44]. The key biological events associated with one potential mechanism linking the MWCNT exposure to the adverse outcome of lung fibrosis were identified. Next, transcriptional BMDs for perturbed biological pathways corresponding to the key events in the hypothetical AOP for fibrosis were calculated using data from dose–response and time-series experiments on rodents exposed to the same three types of MWCNTs [19, 44]. Finally, we compared the PODs derived by NIOSH using traditional risk assessment approaches to transcriptional PODs derived via multiple genomics based approaches (Fig. 1). Our results provide foundational data and guidance to support the use of transcriptional PODs in HHRA of ENMs.

## Methods

### Study design

The present study employed gene expression data from three studies already published in the literature. No additional animal experiments or laboratory experiments were conducted. Details on research conducted on animals and ethics approvals obtained are described in Poulsen et al. [19], Guo et al. [44], and Mercer et al. [34]. In order to derive meaningful data for AOP assessment and HHRA, both time-series and dose–response data are required. We searched the literature for studies that describe transcriptional lung responses to multiple doses of MWCNTs at different post-exposure time-points in a mouse model and found two relevant studies [19, 44]. The analyses were restricted to mouse studies to minimize the loss of information during the statistical normalization of cross-species data and included only those datasets that are publicly available and meet the transcriptomic quality checks recommended by Bourdon-Lacombe et al. [31]. Figure 1 shows the different approaches used in the present study to derive PODs. Additional physico-chemical properties of the three MWCNTs are listed in Additional file 1: Table S1. One study that reported mouse lung fibrotic response to MWCNT by histopathological and morphometric analyses was also included for the purposes of comparison of transcriptomic approaches to apical endpoint-driven risk assessment methods [34]. The study design of the three individual studies considered in the analysis is briefly described below.

- *Study 1*. Poulsen et al. [19] investigated the transcriptomic response of mouse lungs exposed to two types of individual MWCNTs of different lengths: NM-401 (4 ± 0.4 μm in length, European Union Joint Research Centre, Ispra, Italy) and NRCWE-026 (0.8 ± 0.1 μm in length, Nanocyl NC7000 CNT, Sambreville, Belgium). In this study, female C57BL/6 mice were exposed via single intratracheal instillation to 18, 54, or 162 μg/mouse of NM-401 or NRCWE-026 dispersed in MilliQ water with 2 % serum. Control mice were exposed to 2 % serum in MilliQ water only. The selected doses reflect 1, 3, and 9 days of exposure at the Danish eight-hour working day occupational exposure limit of 3.5 mg/m^3^ for Printex90 carbon black particles [45]. Mouse lung tissue was collected 24 h, 3 days, and 28 days post-exposure. Total RNA extracted from the left lung lobe (*N* = 6 per treatment group and time point) was hybridized to Agilent mouse 8 × 60 K oligonucleotide microarrays. LOWESS normalization and MAANOVA analysis was conducted to identify the significantly differentially expressed genes (DEGs). The DEGs with false discovery rate (FDR) adjusted P value of ≤ 0.05 and fold change of ≥ ± 1.5 were used in the downstream analysis by pathway and ontology tools as described in Poulsen et al. [19].
- *Study 2*. Guo et al. [44] investigated the transcriptomic response of male C57BL/6 mouse lungs exposed via pharyngeal aspiration to XNRI MWCNT-7, commonly referred to as Mitsui-7 (3.86 μm in length, Mitsui & Company). Briefly, mice were exposed to a single dose of 10, 20, 40, or 80 μg/mouse of Mitsui-7 dispersed in vehicle (Ca2+ and Mg2+ free phosphate buffered saline supplemented with D-glucose, mouse serum albumin, and 1,2 dipalmitoyl-sn-glycero-3-phosphocholine). Control mice were exposed to dispersion vehicle only. The 10 μg dose used in this study reflects occupational exposure to MWCNTs for a time period between 9 months and 7.5 years based on the average daily MWCNT workplace exposure data for a person performing light work in an environment with MWCNT aerosol of 4–40 μg/m^3^) [46]. Lung tissue was collected 24 h, 7, 28, and 56 days post-exposure. Total RNA extracted from the whole lung (*N* = 8 per treatment group and time point) was hybridized to Agilent mouse 4 × 44 K oligonucleotide microarrays. This microarray dataset has also been used in other studies by Snyder-Talkington et al. [47, 48] and Dymacek et al. [49, 50]. The microarray dataset was downloaded from the GEO database (www.ncbi.nlm.nih.gov/geo) (GSE29042) and was reanalysed employing the same statistical methods as Poulsen et al. [19] above to derive the list of DEGs. Genes showing FDR P value ≤ 0.05 and fold change ≥ ± 1.5 were used in the pathway and ontology analyses.
- *Study 3*. Mercer et al. [34] investigated the pulmonary fibrotic response in male C57BL/6 mice following pharyngeal aspiration of 10, 20, 40, or 80 μg/mouse Mitsui-7. The exposure regimen was the same as the one by Guo et al. [44]. Lung tissues were fixed by intratracheal perfusion of 10 % formalin. The left lobe of the lung was embedded in paraffin, sectioned, and stained with Sirius Red stain for the histopathological and morphometric measurements. The average thickness of Sirius Red positive connective tissue in the alveolar regions was measured using quantitative morphometric methods and the collagen fiber content of granulomatous lesions in the airspaces was assessed as total Sirius Red staining in the airspace.

### Pathway analysis

Functional pathway analysis for classifying genes significantly affected by the exposure to MWCNTs was conducted using Ingenuity Pathway Analysis (IPA, Ingenuity Systems, Redwood City, CA, USA). The canonical pathways consisting of at least five DEGs and exhibiting a Fisher’s Exact P value ≤ 0.05 were considered for further calculation of pathway BMD values for the AOP-dependent approaches.

### Calculation of transcriptional BMDs

Since the purpose of this study was to assess the applicability of toxicogenomic data in quantitative risk assessment for MWCNT-induced lung fibrosis, BMD modeling was conducted on each microarray dataset using BMDExpress version 1.4.1 [51]. Briefly, the BMD analysis was performed on present genes (defined as those that had signal intensities at least three standard deviations above the background non-murine control spots on the array) that showed ANOVA *P* ≤ 0.05 for at least one dose. Four dose–response models; Hill, Power, Linear, and Polynomial (2^0^ and 3^0^) models were used to assess the best fit. Selection of the best fit model required: (a) a nested chi-squared test cut-off of 0.05, (b) the lowest Akaike Information Criterion (AIC), (c) goodness-of-fit P value > 0.1, and (d) the BMD was lower than the highest dose. The benchmark response factor was set to 1.349, which corresponds to a 10 % change compared to controls [51]. The maximum number of iterations, the convergence criteria for model fitting, was set to a maximum of 250, and power was restricted to ≥ 1. If the k parameter of the Hill model was less than one third of the lowest dose used in an experiment [52], the Hill model was flagged and subsequently excluded if at least one other model had a p-value > 0.05. In the case when no other model had a p value > 0.05, the Hill model BMD was adjusted to ½ of the lowest BMD value. The resulting BMDs for each gene were mapped to IPA canonical pathways to assess biological relevance. Median BMD and BMDL (lower confidence limit of BMD) values were calculated for each IPA canonical pathway that had at least five modeled DEGs.

### Calculation of BMDs for apical endpoints

NIOSH conducted an extensive review of the literature pertaining to the human health effects of occupational exposures to both MWCNT and single walled CNTs (SWCNT). BMD analysis was conducted on CNT-induced alveolar septal thickness and other related endpoints reflective of lung fibrosis using the US EPA’s BMD software (BMDS, version 2.1.2) [53]. For dichotomous data, a benchmark response (BMR) of 10 % excess risk was used and for continuous data, a BMR of 1.1 standard deviations above the control mean response was used as an equivalent to a 10 % BMD estimated in dichotomous data. Model fit was considered adequate if goodness of fit P value was greater than 0.05.

## Results and discussion

### Proposed transcriptomics-driven regulatory testing paradigm

Sustained development of products containing ENMs will be impeded by the lack of a pragmatic risk assessment paradigm that can effectively address the unique aspects of nanomaterial risk assessment in a timely manner. Fundamental understanding of the molecular mechanisms underlying toxicity has become a central component of proposed changes to human health risk assessment (HHRA) challenges in the 21^st^ century [9]. Global analyses of perturbations in biological pathways, processes, and functions are playing an increasingly important role in dissecting toxicological mechanisms associated with human health risks. In parallel, increasing emphasis has been placed on dose–response modelling of global transcriptional changes to identify the doses at which critical tissue-level effects begin to occur [54, 55]. Here we propose an integrated approach that leverages advances in high-throughput transcriptomics, dose–response modeling, and international initiatives to develop AOPs of toxicological effects be used to address the important risk assessment gaps for nanomaterials.

In a proof of concept study, we obtained publicly available genomics data from rodent pulmonary exposures to MWCNT for BMD modelling of perturbed genes and pathways. A BMD is the dose associated with a predetermined response level. The statistical lower confidence bound on the BMD (the 95 % lower bound on the BMD) is the BMDL, which is equivalent to traditional No Observed Adverse Effect Level (NOAEL) that is used as a POD. Transcriptional BMDs were linked to key events in a hypothetical AOP for pulmonary fibrosis to assess their potential utility in selecting a POD for nanomaterial HHRA.

**Fig. 2:**
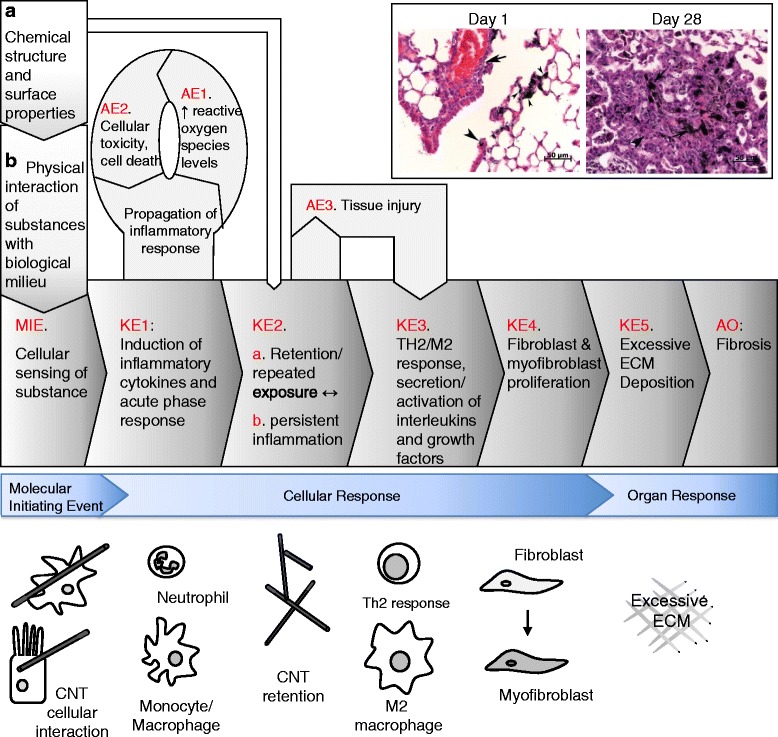
Schematic of adverse outcome pathway (AOP) for pulmonary injury leading to fibrosis. MIE: molecular initiating event, KE: key event, AO: adverse outcome, AE: associative event, CNT: carbon nanotube, ECM: extracellular matrix. Arrows in inset figure show inflammation at day 1 and fibrosis at day 28 in lung tissue

As part of the first steps in deriving transcriptomics-based BMDs for HHRA of MWCNT-induced lung fibrosis, we organised the vast amount of gene expression data derived from the three studies [19, 44] in a systematic and logical manner using an AOP framework. This enabled us to construct a hypothetical AOP describing the various key events (as derived from gene expression and pathway perturbation data) that may occur between the initial exposure to MWCNTs and the development of fibrotic lesions in the lungs (Fig. 2).

The lung constitutes a barrier between the host and the environment. The alveolar capillary membrane (ACM) plays a critical role in gas exchange and is constantly exposed to inhaled or endogenous challenges. Exposure-induced ACM injury and consequent loss of structural integrity results in injury, inciting a repair process that begins with extravasation of plasma into lung tissue, activation of intrinsic and extrinsic coagulation pathways, fibrin deposition, and establishment of a provisional matrix. These steps involve the release of a number of lipid mediators, cytokines, growth factors, and chemo-attractants required for activation of various immune cell types. If the primary insult leading to ACM injury is removed, the deposited extracellular matrix (ECM) is remodelled or reabsorbed. The epithelial and endothelial cells re-establish their normal spatial orientation on the basement membrane of the ACM, restoring the integrity of the ACM, and complete the repair process [56]. However, persistent or recurrent injury from repetitive or persistent exposure to an irritant leads to a deregulated tissue repair process that can result in lung fibrosis. As such, there exists no simplified mechanism that encompasses the pathogenesis of fibrosis in all situations. For example, while fibrosis induced by the inhalation of the high aspect ratio particle asbestos is dependent on inflammatory mediators such as TNF-α [57], cystic fibrosis is driven by a mutation in the CFTR gene and may occur in the absence of inflammation [58]. In idiopathic pulmonary fibrosis (IPF) the specific cause and mechanism is unknown, although exposure to chemicals and particulate matter is deemed a risk factor for IPF [59]. Mechanistic studies that analyzed the targeted inhibition of specific fibrotic mediators are currently not extensive enough to form a complete picture for in vivo models of particulate-induced fibrosis. In general, what we know of pulmonary fibrosis is drawn from the well-characterized murine model of bleomycin-induced pulmonary fibrosis. Bleomycin exposure models outline a process where there is an initial inflammatory response characterized by macrophage- and epithelial cell-derived cytokine production associated with innate immunity. Disruption of these innate immune mechanisms, which include IL-1 and IL-6 signaling, significantly attenuates fibrosis in the bleomycin model [60–62]. This initial response progresses to the activation of CD4+ T cells, which contribute additional cytokines that are crucial to the development of fibrosis [63, 64]. Specifically the Th2 cytokines IL-4, IL-5, and IL-13 that form the adaptive immune response are up regulated, which are key fibrosis mediators [65–68]. Additionally, production of IL-17 from Th17 cells and down regulation of the Th1 response occurs in the fibrotic processes [63, 64, 69]. This activation of an adaptive immune response is independent of antigen sensitization [64], but traditional antigen presenting cells like dendritic cells may contribute to the inflammatory profile [70]. This signaling leads to the production of growth factors, such as TGF-β, which are key regulators of fibroblast activity, and their increased expression disrupts normal homeostatic collagen metabolism, resulting in the excessive collagen deposition in the extracellular matrix characteristic of fibrotic lesions [66, 71]. Other mechanisms, for example, direct stimulation of fibroblasts and epithelial cells with bleomycin can lead to the release of effector molecules [72], which may also contribute to fibrosis in this model.

MWCNTs are non-biodegradable and persist in lung tissues long after the initial exposure, resulting in prolonged tissue injury and unrestricted tissue repair. In addition to their persistence, the high aspect ratio of MWCNTs, their fibre-like structures, and chemical groups on their surfaces are suggested to contribute to the development of lung fibrosis [73], a potential human health effect associated with occupational exposure to MWCNTs [43]. Although several studies have reported lung fibrosis following exposure to MWCNTs, the exact underlying mechanisms are not completely elucidated. Identifying specific mediators and a single mechanism for MWCNT-induced pulmonary fibrosis is challenging. Though data is more limited with respect to MWCNT exposure models, the studies that are available highlight the similarities between the pulmonary response to MWCNTs and bleomycin [33, 74]. MWCNT-induced inflammation, as indicated by neutrophil influx into the lungs, is dependent on IL-1 signaling through the IL-1 receptor 1 (IL-1R1) [75], which involves the activation of the NLRP3 inflammasome [76]. The characteristics of the inflammatory response induced by MWCNTs parallels the bleomycin model, which is reflected by the induction of Th2-like phenotype, involving the production of cytokines such as IL-13 [77, 78]. The inflammatory response ultimately leads to the alteration of fibroblast activity resulting in enhanced activity of TGF-β, which is also implicated in MWCNT-induced fibrosis [79]; the TGF-β/SMAD pathway is involved in fibroblast differentiation and epithelial mesenchymal transition; another characteristic of fibrotic lesions [80]. Additional factors such as the generation of reactive oxygen species have been shown to contribute to fibrotic pathogenesis; animals deficient in anti-oxidant responses elaborate exacerbated fibrosis in response to MWCNTs [81]. While results from in vivo models described in the present study suggest a role for pulmonary inflammation in MWCNT-induced lung fibrosis, many in vitro studies have suggested a plausible role for direct action of CNTs (both MWCNTs and SWCNTs) on fibroblast function and viability [77, 82–88] that may lead to fibrosis. Moreover, these studies demonstrate increased fibroblast proliferation, collagen synthesis, and expression of the fibrosis-associated gene *Mmp9*, all of which serve as in vivo markers of lung fibrosis [77, 85–88]. However, these findings have not been confirmed in vivo and additional research as to the relevance of the direct stimulation of fibroblasts in isolation of all of the other resident cells of the lung is necessary. Thus, similar to the bleomycin model, multiple mechanisms likely contribute to MWCNT-induced fibrosis but the focus on the inflammatory response triggered by MWCNTs and the resulting pathology from these pathways is warranted given the data generated from models of MWCNT exposure.

It is important to note that other signaling pathways not directly related to innate and adaptive immune mechanisms may also contribute to MWCNT-induced fibrosis (such as direct stimulation of fibroblasts by CNTs), but the focus on the inflammatory response triggered by MWCNTs and the resulting pathology from these pathways is warranted given the crucial role that inflammation is known to play in other models such as bleomycin-induced or Th2-response triggered fibrosis.

Thus, based on the literature review of the general mechanisms involved in lung fibrosis (described above), and comprehensive analyses of gene expression profiles perturbed following exposures to different doses of MWCNTs at different post-exposure time-points, a hypothetical AOP for pulmonary fibrosis was proposed according to the guidance set by the Organisation for Economic Cooperation and Development (OECD) [89–91]. The AOP that we describe is a purposefully simplified linear pathway constructed for the purposes of organising the mechanistic data derived from the global gene expression studies, and to facilitate selection of key pathways and events to use for POD calculation. A full description of the AOP, with the weight of evidence supporting the key event relationships according to the modified Bradford-Hill criteria described in the AOP Users’ Handbook, is beyond the scope of this manuscript and is under development as part of the OECD’s AOP program.

Thus, the putative AOP consists of a molecular initiating event (MIE) and key events (KEs) vital to the development of lung fibrosis (Fig. 2). Although the networks and biology involved in the development of lung fibrosis are very complex, two criteria consistent with the OECD guidelines were used in constructing the AOP: 1) the MIE and KEs were based on their essentiality to the initiation and progression of lung fibrosis (i.e., inhibition of occurrence of these KEs would influence the final fibrosis outcome); and 2) the MIE and KEs had to be measurable/quantifiable. It is important to note that the putative AOP described below is simplistic and reflects only one potential mechanism, by which MWCNTs induce lung fibrosis. We agree that there may be several converging pathways that are involved in lung fibrosis. However, the AOP program itself acknowledges that AOPs are purposefully over-simplified linear pathways to toxicity (http://aopkb.org/common/AOP_Handbook.pdf). The aim here is to develop simple linear pathways that address contained aspects of a pathway to toxicity. The AOPs should contain only key events that are routinely measured (and are essential), and subsequent to that the simplified linear AOPs provide a basis to develop broader networks that can be used in predictive toxicology. Accordingly, the AOP for lung fibrosis induced by MWCNTs consisted of the following steps:

- MIE – Cellular sensing of the substance-induced damage resulting in the release of danger signals.
- KE1 - Induction of inflammatory cytokines and acute phase response. This KE involves the recruitment and infiltration of various immune cell types into the lung tissue, which will clear the invading substance and initiate the tissue repair process. This step is commonly known as acute inflammation. Although a long list of cytokines and chemokines are induced during the acute inflammatory phase, since MWCNT-induced acute inflammation is predominantly characterised by neutrophil infiltration, we argue that IL-1R1-mediated pathway is primarily involved in this process. Thus, secretion of IL-1R1-regulated cytokines and chemokines including IL1, IL6, CXCL-1, CCL2, CCL5, and SAA3, as well as IL1β, can be used to measure KE1.
- KE2 – a) Retention or repeated exposure and b) persistent inflammation. The inability to clear MWCNTs results in ‘frustrated phagocytosis’ leading to prolonged interaction of MWCNTs with the lung epithelium, continuous activation of stress signalling, inflammatory response, and unremitting repair process. The ensuing chronicity of inflammation engages the adaptive immune system, which then results in the third KE. Both KE 2a and 2b act in a positive feedback loop and are essential for the activation of KE3. Although ‘frustrated phagocytosis’ is suggested to occur in response to specific-types of long MWCNTs, other types of short or entangled MWCNTs including NM-401 and NRCWE-026 also biopersist, which can similarly result in chronicity of inflammation leading to disruptive repair process.
- KE3 - TH2/M2 response, secretion/activation of interleukins and growth factors. This KE involves activation of T helper (Th) 2 type cells and the release of specific cytokines necessary for activation of M2 type macrophages. M2 macrophages secrete anti-inflammatory cytokines and tissue inhibitors of metalloproteinases that impair remodelling or reabsorption of the deposited ECM. Several TH2 type cytokines including IL-4, IL-5, IL-13 and growth factors such as transforming growth factor beta, that play an important role in the progression of lung fibrosis are upregulated following exposure to MWCNTs and can be used to quantify the activation of KE3.
- KE4 – Fibroblast and myofibroblast proliferation. The late stages of tissue injury involve chronic inflammation, epithelial and endothelial injury, loss of ACM integrity, and activation and proliferation of fibroblasts/myofibroblasts.
- KE5 – ECM deposition. The repeated cycles of the preceding KEs result in excessive ECM deposition due to imbalance between the deposition and metabolism of ECM proteins, including collagen.
- AO – fibrotic lesions. Excessive ECM deposition results in the development of lung fibrotic lesions (Adverse Outcome - AO).

In addition to the KEs described above, associative events (AEs) occur in parallel and can enhance the probability that the exposure will lead to the AO. In this AOP, the continual release of soluble mediators produced by the activated inflammatory cell types, and subsequent interaction between the cell types, leads to increased reactive oxygen species levels (AE1, oxidative stress) and cellular apoptosis (AE2), which contribute to repeated tissue injury (AE3). The other associative events such as, resistance of fibroblasts to apoptosis, collagen metabolism, or inhibition of collagen-degrading enzymes may play a regulatory role in the progression of lung fibrosis. Thus, these events are not included in the hypothetical AOP presented and will be included in a separate AOP.

The putative AOP as described above was subsequently used to categorize and prioritize the significantly perturbed pathways and to calculate the median BMD(L) values of genes within those pathways to derive pathway-based PODs in an AOP-dependent transcriptomics approach (Fig. 1). The KE-associated BMDs were compared with BMDs derived from analysis of conventional toxicology endpoints (e.g., an apical endpoint associated with fibrosis), and to AOP-independent transcriptional approaches, to explore the best approach to selection of a POD.

In our previous studies, we have characterised transcriptomic changes occurring in lungs of mice exposed to carbon black (CB) or titanium dioxide nanoparticles (TiO_2_NPs). In order to determine if the MIE and KEs in the putative AOP described above for MWCNT-induced lung fibrosis are also observed following exposure to other control particles, such as CB or TiO_2_NPs, we compared the pathways reported to be significantly perturbed following CB [22, 92] or TiO_2_NP [16] exposures to those perturbed following exposure to MWCNTs. The results showed that exposure to CB or TiO_2_NP induces gene expression changes associated with lung inflammation, Acute Phase Response Signaling and Chemokine Signaling pathways [16, 22, 92] that are relevant to MIE and KE1. However, it was noted that the number of genes perturbed in each pathway was few and the relative fold-changes were smaller compared to the MWCNT-induced response. Moreover, most of the significantly perturbed genes did not show dose–response (data not shown), as a result they could not be modeled using BMDExpress. These results suggested that although CB and TiO_2_NP induce lung inflammation and induce altered expression of genes and pathways associated with MIE and KE1, the pro-fibrotic events described in KE2- 4 and AO are specific to MWCNTs. Thus, control particles were excluded from the rest of the analysis in the present study.

### Functional analysis of perturbed genes and dose–response modeling

The BMD(L) values for all of the genes were calculated, the genes were assigned to pathways, and median pathway BMDs were thus calculated for each time-point and for each MWCNT. The 3 days Poulsen et al. [19] and 7 days Guo et al. [44] time-points perturbed similar pathways and since they represent early, acute post-exposure time-points, they were considered together. This also implies that the pathways leading to lung fibrosis are activated as early as 3 days post-exposure; however, the clinical manifestation as measured by alveolar thickness may become apparent on 7 days after the last exposure [93] (reviewed in [94]).

The lists of differentially expressed genes (DEGs) derived from Poulsen et al. [19] and Guo et al. [44] were analyzed for functional significance using IPA. The significantly perturbed pathways (*P* < 0.05) were categorized according to their association with the KEs and AEs in the AOP for lung fibrosis. Pathways for which the biology did not appear to be related to the KEs or AEs in the AOP were not included in the analysis. A few pathways overlapped with more than one KE: Chemokine Signaling (KE1, KE3), Dendritic Cell Maturation (KE1, KE3), Jak/Stat Signaling (KE1, KE3), Leukocyte Extravasation (KE1, KE2), NF-kB Signaling (KE1, KE2), and TGF-beta Signaling (KE3, KE4). The complete list of perturbed pathways and the associated BMDs can be found in Additional file 2: Table S2. Unique biological pathways were perturbed at different post-exposure time-points with some overlap. For example, Acute Phase Response Signalling and Hepatic Fibrosis/Hepatic Stellate Cell Activation pathways were significantly enriched at the 24 h, 3, and 28 days time-points for NM-401. There is no separately annotated pathway for lung fibrosis in IPA. The “Hepatic Fibrosis/Hepatic Stellate Cell Activation” pathway includes pulmonary fibrosis in the IPA pathway analysis tool. Fibrosis is a wound healing process characterized by accumulation of extracellular matrix and the components of ECM accumulation are similar regardless of the cause or tissue. Thus, for the rest of the discussion, this pathway will be referred to as the ‘Fibrosis’ pathway. A large number of biological pathways associated with the KEs were perturbed 3 days post-exposure for all three types of MWCNTs investigated. Thus, gene expression profiling supports that the post-exposure time-points at which the KE is observed has a strong influence on whether it is measurably perturbed, which can be used to support the temporal order of events.

**Fig. 3:**
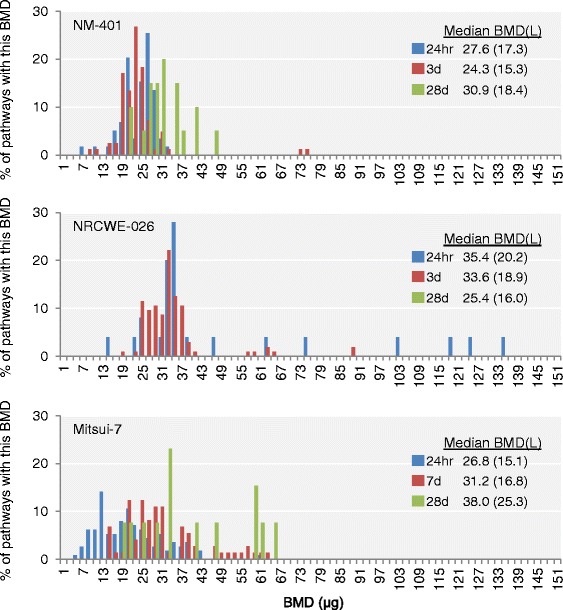
The distribution of pathway BMD-median values is influenced by post-exposure time. Distributions of pathway BMD-median values for NM-401 (*top*), NRCWE-026 (*center*), and Mitsui-7 (*bottom*). Pathways were only considered in this analysis if they were significant (*P* < 0.05) with five or more DEGs associated with them and if they had five or more molecules with goodness-of-fit P value > 0.1 and BMD/BMDL ratios < 10. Overlain table indicates the median BMD(L) across all pathways for each time-point

In general, analysis of the distribution of pathway BMDs revealed that most pathway BMD(L) values (Fig. 3), including those associated with KEs, were lower at the earlier time-points (24 h, 3 days) for NM-401 and Mitsui-7. In contrast, NRCWE-026 had similar BMDs at 24 h and 3 days, but had very few pathways perturbed at the 28 days time-point and only one KE-associated BMD that could be derived for this time-point (discussed below). Overall, some of the lowest BMD(L) values were for pathways that were associated with more than one KE and that were perturbed across MWCNTs and time-points, including Chemokine Signaling, Dendritic Cell Maturation, and Jak/Stat Signaling. The BMD(L) values were remarkably similar overall across different time-points and MWCNT types. However, a few exceptions were noted where BMD(L) values were higher for the pathways perturbed at the later post-exposure time-points (28 days), suggesting a time-dependent transition in the toxicity pathways and that the pathways implicated in the pathological manifestation of the disease occur at the later time-points. Thus, although different pathways were altered at different post-exposure time-points, the BMD(L) values within a KE were consistent across time-points and particle types.

**Fig. 4:**
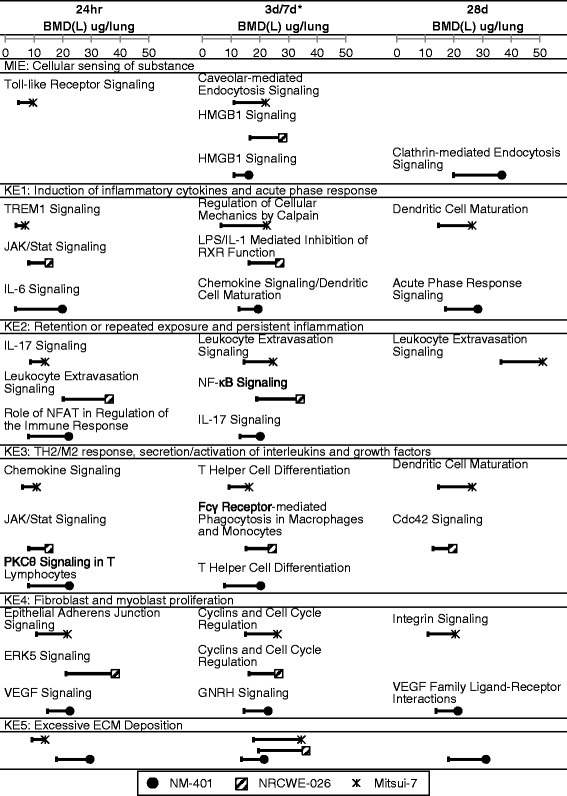
Pathways with the lowest BMD(L) values for each part of AOP per MWCNT and time-point. Figure shows the pathway with the lowest BMDL for the MIE (Molecular Initiating Event) and each KE (Key Event) for NM-401 (*solid black circles*), NRCWE-026 (*square with hatched lines*), and Mitsui-7 (*star*). The 95 % lower confidence interval (BMDL) for each BMD is represented by the error bars. Only significant canonical pathways (*P* < 0.05, >5 genes) with BMD/BMDL ratios <10 and >5 genes modeled were included. The 3d/7d time-point with the asterisk (*) is a placeholder for 7 days for Mitsui-7 as there was no 3 days time-point recorded. Blank spaces indicate no pathway was significant for that time-point. The KE5 only includes the pathway Fibrosis

Interestingly, similar pathways were perturbed following exposure to the three types of MWCNTs across the time-points, despite differences in their physico-chemical properties, suggesting that the underlying mechanisms leading to fibrosis are the same for all three MWCNTs (Additional file 2: Table S2). For example, HMGB1 Signaling, NF-kB Signaling, and Dendritic Cell Maturation were perturbed at 3 days by NM-401 and NRCWE-026 and at 7 days by Mitsui-7. In addition, Acute Phase Response was perturbed at 24 h, 3, and 28 days by NM-401 and Agranulocyte Adhesion and Diapedesis was perturbed at 24 h, 7, 28, and 56 days by Mitsui-7. Additionally, although the magnitude of changes in the expression levels of the individual genes differed between the MWCNT types [19, 44], which was reflected in the subtle differences between the BMD(L) values for a specific pathway for individual MWCNT types, the BMD(L) confidence intervals across the MWCNT types overlapped (Fig. 4). NRCWE-026 significantly altered a single pathway 28 days post-exposure (CDC42 Signaling). In addition, the pathway BMD(L) values 3 days post-exposure were relatively higher for this MWCNT than BMDL values at 3 days for NM-401 or at 7 days for Mitsui-7. BMDL values ranged from 15.1 to 21.2 μg/mouse for NRCWE-026, 8.1–18.4 μg/mouse for NM-401, and 7.1–30.3 μg/mouse for Mitsui-7. The lack of significant pathways at 28-days post-exposure to NRCWE-026 corroborates histopathological observations in the lungs: qualitative assessment of the lung sections from mice exposed to NRCWE-026 showed fewer incidences of fibrotic lesions 28 days post-exposure than NM-401, as described in Poulsen et al. [19]. Additional file 3: Figure S1 depicts a heat map revealing the dose at which each pathway was significantly perturbed. Given that all three MWCNTs perturbed similar pathways across doses and time-points, the results can be broadly generalized to understand the potential lung responses induced by similar nanomaterials, and the approach can be used as a sensitive alternative for read across purposes and to identify properties that are important for material toxicity.

### Apical endpoint BMD(L)s

NIOSH conducted an extensive review of the literature pertaining to the human health effects arising from occupational exposures to both MWCNT and SWCNT [43]. BMD(L)s for MWCNTs were derived using fibrosis-related apical endpoint data (e.g., alveolar connective tissue thickening, alveolar septal thickening, granulomatous inflammation, and hydroxyproline amount) presented in four individual studies [32–35] (Additional file 4: Table S3). Only data from Mercer et al. [34] were included in the present study for comparison of apical and transcriptomic PODs because: (a) the experimental design and the MWCNT studied was identical to Guo et al. [44], which investigated the pulmonary transcriptomic response; and (b) it is the only study that examined apical endpoints in a mouse model, which minimizes cross-species extrapolation. Morphometric analyses of histological sections for alveolar connective tissue thickening, a biologically relevant event in pulmonary fibrosis, were used in Mercer et al. [34] to calculate apical endpoint BMD(L) values and to derive a POD. This analysis revealed BMD and BMDLs of 27.1 and 14.1 μg/mouse, respectively for lung fibrosis induced by Mitsui-7.

### Approaches for deriving transcriptional PODs

Several different approaches were explored to determine the best method to derive a transcriptional POD: 1) the lowest BMD(L) value for any pathway, 2) the median pathway BMD(L) value, 3) the lowest BMD(L) value based on pathways related to the KE determined to be the ‘point of no return’ (the initiation/occurrence of a specific key event, beyond which the removal of the toxic substance or inhibition of injury does not result in the reversal of effects or recovery), and 4) the BMD(L) value based on pathways related to lung fibrosis. These approaches include AOP-independent (1 and 2) and AOP-dependent approaches (3 and 4) for deriving a transcriptional POD.

**Fig. 5:**
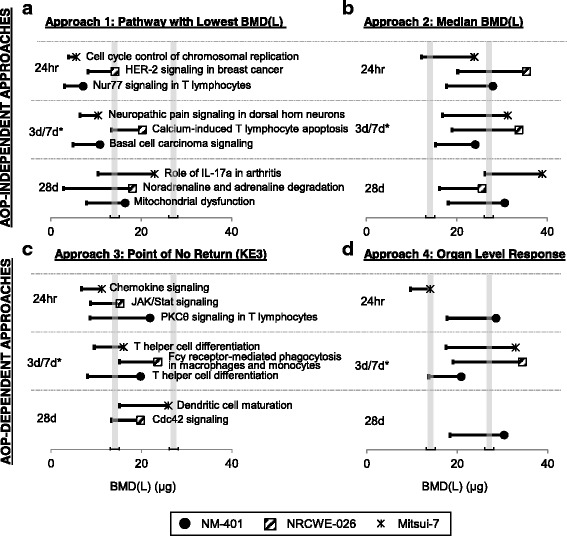
Comparison of BMD(L)s derived from four genomics approaches compared to the traditional NIOSH approach. Both AOP-independent (approaches 1 and 2; panels (**a**) and (**b**), respectively) and AOP-dependent approaches (approaches 3 and 4; panels (**c**) and (**d**), respectively) are shown. The grey lines represent the BMD (*right bar*) and BMDL (*left bar*) values for fibrosis apical endpoint [34, 43]. BMD values are represented by solid circles for NM-401, hatched squares for NRCWE-026, and stars for Mitsui-7. The 95 % lower confidence interval (BMDL) for each BMD is represented by the error bars. The 3 days/7 days time-point with the asterisk (*) is a placeholder for 7 days for Mitsui-7 as there was no 3 days time-point recorded

Approach 1, based on the most sensitive pathways (Fig. 5a), yielded BMD(L)s between 7.1 (3.0) and 14.2 (8.2) μg/mouse for NM-401, 10.9 (4.9) and 20.2 (13.4) μg/mouse for NRCWE-026, and 18.0 (2.8) and 23.0 (10.43) μg/mouse for Mitsui-7 across all post-exposure time-points. For the most part, the BMD(L) values for these pathways were below the NIOSH-calculated BMD(L) of 27.1 (14.1) μg/mouse, which was based on measuring alveolar thickness [34, 43]. The most sensitive pathway approach has been recommended for deriving PODs for cancer and non-cancer endpoints [28]. Although the pathways may be reflective of the tissue injury caused by the initial interaction of MWCNTs with the surrounding lung tissue, they were not consistently observed across the types of MWCNTs, doses, or post-exposure time-points. Moreover, their implication in the final adverse outcome is less understood. Indeed, a POD based on the most sensitive or lowest BMD(L) values may not be based on a biological effect that is connected to the adverse outcome. However, we note that there are very few nanomaterials for which underlying mechanisms of toxicity are characterized or a definite adverse outcome has been identified. Thus, while this AOP-independent approach may be too conservative, it would serve to limit the exposure to particular nanomaterials in the environment until specific hazard and mechanistic information becomes available for them.

The median BMD(L) value approach, which is also AOP-independent (based on BMDs for all pathways with five or more DEGs associated with them and if they had five or more molecules with goodness-of-fit P value > 0.1 and BMD/BMDL ratios < 10), represents a less conservative approach to POD selection (Fig. 5b). For the most part, the BMD(L) values using this approach were above the NIOSH-calculated BMD(L) of 27.1 (14.1) μg/mouse with the exception of a few values (23.9 (15.1) μg/mouse for Mitsui-7 at 24 h, 24.1 (15.3) μg/mouse for NM-401 at 3 days, and 25.6 (16.0) μg/mouse for NRCWE-026 at 28 days). However, the confidence intervals of the median values (the median is the peak of biological activity and represents the most robust central measure of the response) were very close to the lower confidence intervals of the NIOSH-calculated BMD and BMDL values. Thus, without prior knowledge of the underlying mechanism of toxicity or a definite adverse outcome, as is the case with nanomaterials, this approach provides a point at which the system is likely to be responding to the exposure.

Transcriptional BMDs based on the AOP-dependent approaches (approaches 3 and 4; Fig. 5c-d) resulted in values consistent with the conventional fibrosis endpoint (alveolar wall thickness, represented by grey bars in Fig. 5). Of benefit in these approaches is the fact that these pathway perturbations are biologically linked to the AO, and thus it is biologically plausible that the doses that affect these pathways might approximate the dose at which adversity may arise. Modes of Action (MOAs) have been routinely employed to inform risk assessments for chemicals. However, MOA in the conventional sense provides information concerning molecular and cellular events engaged by the chemicals in eliciting a response that is quantified but may or may not always imply that the response will induce an adverse outcome. AOPs have evolved from the concept of MOAs, and also emphasize the importance of connecting various events that lead to toxicity. Both require selection and ordering of the essential events that are readily quantifiable along the pathway to toxicity, and require assessment of empirical data (temporal and incidence concordance) and biological plausibility supporting the relationship shared between events along the pathway. In approaches 3 and 4, perturbed pathways were characterized in terms of their association with KEs, which provides a biological rationale for their consideration in POD selection (if apical data are not available). In approach 3, we selected a single KE (KE3: Th2 Signaling activation), the activation of which we argue marks the ‘point of no return’ in the process of developing lung fibrosis. The various pathways involved in KE3 including Dendritic Cell Maturation, Cdc42 Signalling, T Helper Cell Differentiation, Fcy Receptor-Mediated Phagocytosis in Macrophages and Monocytes, Chemokine Signalling, JAK/Stat Signalling, and PKC Theta Signalling in T Lymphocytes, showed BMDs that spanned the POD range published by NIOSH for the apical endpoint of concern (alveolar connective tissue thickening) (Fig. 5c). In approach 4, the POD selection was based on a pathway tied directly to the AO, which is the lung fibrosis pathway described under KE5. However, in our case, the BMDs based on the pathways associated with fibrosis (the ultimate result of exposure to MWCNTs) were less consistent for the three MWCNTs and across the different exposure time-points (Fig. 5d) relative to the other three approaches. The inconsistencies may be due to the fact that representative pathways for fibrosis were not significant across the MWCNT types, doses and post-exposure time-points.

Taken together, the four approaches for transcriptional- or pathway-based POD derivation produced comparable results across time-points and across the three types of MWCNTs. The confidence intervals between datasets often overlapped; however, the AOP-centered approach based on the ‘point of no return’ KE (approach 3) and the median BMD approach (approach 2) more closely reflected the histopathological observations demonstrating alveolar thickness that are critical for the development of fibrosis.

It is important to note that although the individual MWCNTs varied in their physico-chemical properties, all of them bio-persist long after the exposure and exhibit potential to induce lung fibrosis. In addition, at the molecular level, they induced similar pathway perturbations and BMD(L) values suggesting that the mechanisms underlying the fibrotic responses are the same for the three MWCNTs. The fact that all three MWCNT types induce similar response at the pathway level is interesting and suggests that all MWCNTs are inherently capable of inducing lung fibrosis, which may be associated with their fiber-like shape and tissue biopersistence. However, some MWCNTs may be more potent than others in inducing lung fibrosis, which could be attributed to their additional physico-chemical properties such as chemical impurities, surface functionalization, etc. Previously, we have shown that subtle differences in the degree of pathway perturbation somewhat reflects the extent of fibrosis development [19]. In another study, we have shown that changes in the gene expression levels and pathway perturbations can effectively discriminate between the nanoTiO_2_ particles that are weak or strong inducers of lung inflammation [16]. Collectively, these results suggest that transcriptomic responses are a sensitive approach to identifying potentially harmful nanomaterials and that the transcriptomic BMD(L)s can be used to establish acceptable levels of exposure for new nanomaterials. As the public repository of genomics dataset is populated and the underlying mechanisms of toxicity are revealed, these types of data can be used in read across and category approaches for risk assessment.

## Conclusions

In conclusion, the results show that transcriptomics-derived PODs accurately reflect the levels of exposure to MWCNTs at which lung fibrosis potentially develops in animals. The significant overlap in pathway responses induced by the different types of MWCNTs and comparable pathway-based POD values suggests that the approach can be generalised to other forms of MWCNTs. This study demonstrates that in the absence of any apical endpoint toxicological data conventionally preferred in HHRA, transcriptomics data can be used to inform the potential hazards of these novel materials and to support interim regulatory decisions. We speculate that this toxicogenomics/AOP approach will be of value for prediction of fibrogenic potency and hazard ranking of different MWCNTs. The case study is limited to the analysis of in vivo genomics data and its applicability to HHRA, but we emphasize that this approach does not represent a full risk assessment. While analysis of a well-characterized nanomaterial known to induce adverse effects provides an initial framework for future studies, regulatory validation of these methods will require a large repository of publicly available high quality gene expression data that span several well-characterised reference nanomaterials of diverse properties, multiple doses, a range of post-exposure time-points, and multiple species. Guidance and appropriate criteria for assessing experimental design and quality of data produced, rigorous statistical and computational algorithms to handle the large amount of data, and harmonised guidelines for integrating omics data into HHRA are needed. Finally, given the complexity of data, education and training programs for researchers and risk assessors in the generation, analysis, and application of omics data to different components of the risk assessment paradigm should be developed.

## Additional files

Additional file 1: Table S1. Particle physicochemical properties and exposure information. (XLSX 10 kb)

Additional file 2: Table S2. List of all significant pathways associated with the molecular initiating event (MIE), each key event (KE), and each associative event (AE) including the benchmark dose (BMD) value (BMDL values included in parentheses) for each MWCNT investigated at each time-point. n.a. indicates the BMD(L) could not be calculated due to poor model fit. (XLSX 18 kb)

Additional file 3: Figure S1. Heatmap showing the dose at which each pathway was significantly perturbed. Each column represents a post-exposure time point for the denoted MWCNT, and each row represents a signifncatly perturbed pathway. All colored cells represent the lowest dose at which the pathway is perturbed. Blank cells represent pathways that were not significantly perturbed. (PPTX 76 kb)

Additional file 4: Table S3. Details of rodent studies considered by NIOSH in calculating points of departure (PODs). (DOCX 15 kb)
