# Supplementary figures and images for: Nano-risk Science: application of toxicogenomics in an adverse outcome pathway framework for risk assessment of multi-walled carbon nanotubes
Source: Part Fibre Toxicol. 2016 Mar 15;13:15. doi: 10.1186/s12989-016-0125-9 (PMC4792104; doi:10.1186/s12989-016-0125-9)

## Slide 1
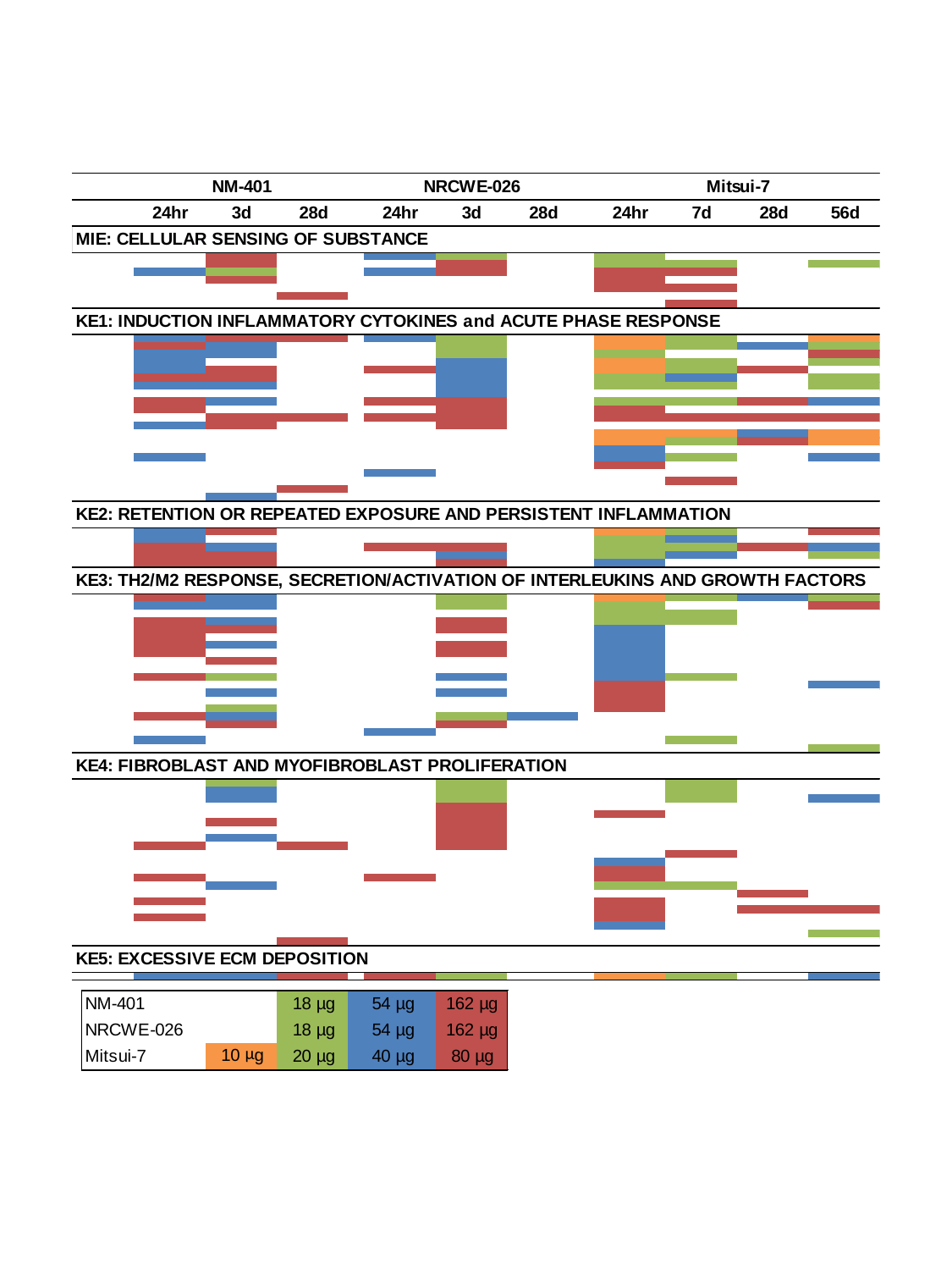

Supplement: Supplementary file 3 — Heatmap showing the dose at which each pathway was significantly perturbed. Each column represents a post-exposure time point for the denoted MWCNT, and each row represents a signifncatly perturbed pathway. All colored cells represent the lowest dose at which the pathway is perturbed. Blank cells represent pathways that were not significantly perturbed. (PPTX 76 kb) [file 12989_2016_125_MOESM3_ESM.pptx]
