# Supplementary material for: Nano-risk Science: application of toxicogenomics in an adverse outcome pathway framework for risk assessment of multi-walled carbon nanotubes
Source: Part Fibre Toxicol. 2016 Mar 15;13:15. doi: 10.1186/s12989-016-0125-9 (PMC4792104; doi:10.1186/s12989-016-0125-9)
Supplement: Supplementary file 4 — Details of rodent studies considered by NIOSH in calculating points of departure (PODs). (DOCX 15 kb) [file 12989_2016_125_MOESM4_ESM.docx]

**Additional file 4: Table S3**. Details of rodent studies considered by NIOSH in calculating points of departure (PODs).

|  | **Mercer et al., 2011** | **Pauluhn et al., 2010** | **Ma-Hock et al., 2009** | **Muller et al., 2005** |
| --- | --- | --- | --- | --- |
| Species | Mouse | Rat | Rat | Rat |
| Strain | C57BL/6 | Wistar | Wistar | Sprague-Dawley |
| Route of exposure | pharyngeal aspiration | inhalation^a^ | inhalation^a^ | intratracheal instillation |
| Dose | 0, 10, 20, 40, 80 µg | 0, 0.1, 0.45, 1.62, 5.98 mg/m^3^ | 0, 0.1, 0.5, 2.5 mg/m^3^ | 0, 0.5, 2.5 mg |
| Post-exposure time (days) | 1, 7, 28, 56 | 1, 28, 91, 182 | 1 | 28, 60 |
| Lung response | Alveolar connective tissue thickness | Alveolar septal thickening | Granulomatous inflammation | Hydroxyproline amount |
| BMD(L) | 27.1 (14.1) µg | 28.0 (14.0) µg^b^ | 21.0 (8.1) µg^b^ | 760 (486) µg |

^a^ inhalation exposures took place for 6hr/d, 5d/wk, for 13wk.

^b^ BMD estimates calculated for grade 1 or higher severity of lung responses
